# A study protocol for a randomized controlled feasibility trial of behavioural therapy for interepisode bipolar symptoms (STABILISE)

**DOI:** 10.1186/s40814-025-01678-6

**Published:** 2025-07-10

**Authors:** Kim Wright, Fiona Warren, Sandra Bucci, Barnaby D. Dunn, Steven Jones, Heather O’Mahen, Rod S. Taylor, Antonieta Medina-Lara

**Affiliations:** 1 Department of Psychology, University of Exeter, Exeter, UK; 2 Exeter Clinical Trials Unit, University of Exeter School, Exeter, UK; 3 Division of Psychology and Mental Health, The University of Manchester, Manchester, UK; 4 Greater Manchester Mental Health NHS Foundation Trust, Manchester, UK; 5 Faculty of Health and Medicine, Spectrum Centre for Mental Health Research, Lancaster University, Lancaster, UK; 6 MRC/CSO Social and Public Health Sciences Unit & Robertson Centre for Biostatistics, School of Health and Well Being, College of Medical, Veterinary and Life Sciences, University of Glasgow, Glasgow, UK; 7 Department of Public Health and Sport Sciences, University of Exeter, Exeter, UK

**Keywords:** Bipolar disorder, Cyclothymic disorder, Behavioural therapy, Psychological therapy, Interepisode symptoms

## Abstract

**Background:**

In between episodes of (hypo) mania and major depression, people with bipolar disorder can experience ongoing low mood or mood instability, and these may also be present as part of cyclothymic disorder. This is a phase II evaluation of an adapted form of behavioural therapy (STABILISE) for inter-episode bipolar symptoms. The study aims to establish the feasibility and acceptability of the therapy and research procedures, including an economic component, to inform a future definitive trial.

**Methods:**

Patients will be randomised 1:1 to either Treatment as Usual (control arm) or Treatment as Usual plus STABILISE intervention (intervention arm). Follow up points will be at 14, 30 and 52 weeks post eligibility confirmation, with 30 weeks as the primary end point. We aim to recruit 60 individuals meeting diagnostic criteria for a Bipolar Spectrum Disorder, and reporting ongoing bipolar symptoms (low mood or mood instability) outside of a manic or severe depressive episode. Feasibility and acceptability will be examined through recruitment and retention rates, completion rates for the candidate primary outcome measures (PHQ9, ALS-SF, QoL.BD and BRQ) and feedback from participants on their experience of study participation and therapy. Proceeding to a definitive trial will be indicated if the following criteria are met: (i) trial participation is deemed, or can be made, sufficiently safe; (ii) recruitment rate indicates that larger-scale recruitment would be feasible (recruitment rate of at least two participants per month within at least one site, with mitigation plan if overall target sample size not met); (iii) for candidate primary outcome measure follow up data is available at 30 weeks from at least 75% of participants, or from between 55 and 74% with clear plan for improvement.

**Discussion:**

This study is a randomised, controlled feasibility trial that builds on an initial case series of the STABILISE approach. The findings will be used to establish whether a future, definitive trial is feasible and to refine the research procedures and therapy protocol.

**Trial registration:**

ISRCTN18207465. Registered 13th March 2024, https://www.isrctn.com/ISRCTN18207465.

**Supplementary Information:**

The online version contains supplementary material available at 10.1186/s40814-025-01678-6.

## Background

Bipolar spectrum disorders (Bipolar I or II Disorder, Cyclothymic Disorder) result in substantial personal and societal costs and affect around 1 in 20 people across their lifetime [1, 2].

In addition to major episodes of depression or mania, people with bipolar spectrum disorders can experience ongoing bipolar symptoms (inter-episode Bipolar symptoms (IEBS)). With IEBS, depressive symptoms tend to be more frequent relative to hypomanic symptoms [3] and instability of mood is common [4].

Interventions are needed for IEBS for three reasons. First, they are common: ongoing bipolar symptoms in the form of subclinical low mood or mood instability are experienced by up to half of those with Bipolar I or II Disorder who are not in a major episode [5], and on average people with bipolar I or II disorder spend around twice as long experiencing residual symptoms as they spend in acute episodes [3]. Second, they are associated with significant distress and impairment, including increased psychiatric comorbidity and poorer functioning [4–9]. Third, the ongoing mood instability that constitutes cyclothymic disorder is associated with risk of developing full depression and mania [10], which can be costly to the health service and the wider society, and costly to patients and families [2].

Despite the impact of IEBS, studies evaluating approaches to helping people with this disabling presentation are scarce. Furthermore, whilst some have examined the effects of pharmacological agents on residual symptoms [11], relatively few consider their impact on mood instability.

Psychological therapies for individuals with bipolar disorder are valued by service users, and are recommended within various national treatment guidelines, yet the extant literature does not provide direct guidance on the optimal psychological treatment for people with IEBS, with studies tending to focus on relapse prevention or alleviation of acute depression as the primary target of therapy [12] or specifically upon cyclothymic disorder [13]. Thus, we do not currently have a psychological treatment that seeks to address the common ongoing mood symptoms (subclinical low mood and mood instability) that people with bipolar disorder (BD) or cyclothymic disorder (CD) present with, nor a therapy that has been tested across the bipolar spectrum with respect to these issues.

Our approach (STABILISE: Study of Therapy for Bipolar Interepisode Symptoms) brings together two behavioural therapies that are commonly used and effective with related populations to address key therapy targets also relevant to IEBS: reduction of depressive symptoms through supporting meaningful behaviours, and management of problematic affective instability. The resulting therapy is designed to support people with BP or CD in managing ongoing difficulties with low mood or mood instability, including the impact of these on everyday life.

Behavioural activation (BA) is used extensively in the treatment of acute unipolar depression [14, 15]. It is informed by the behavioural theory of depression whereby depression is hypothesised to be maintained by a reduction in access to positive reinforcement from the person’s context, and a subsequent increase in ‘avoidance’ behaviours that function to reduce negative affect rather than to approach valued goals. Correspondingly, BA involves supporting the person to access previous and new sources of reward through scheduling goal and value-consistent activities into the week and learning or re-accessing skills for managing barriers to engaging fully in these activities. BA is often considered to represent a parsimonious and relatively straightforward approach to disseminate, potentially reducing training costs and broadening the range of health professionals who may be able to deliver it [16]. Two reports of case series data on its use in people with acute bipolar depression [17, 18] describe high acceptability rates, no safety concerns and encouraging patterns of change on clinical outcome measures.

Dialectical behaviour therapy (DBT) is a form of behavioural therapy originally developed for women with borderline or emotionally unstable personality disorder [19, 20] and later applied across genders and across multiple problem areas. It is based upon the biosocial theory which posits that emotion dysregulation is the outcome of an interaction between an emotionally sensitive temperament and an early environment that invalidates their emotional responses. DBT teaches the person skills for recognising and responding to emotional states, and for managing the situations that can trigger these responses. A behavioural framework is used to understand the interaction between the person and their context, and the therapist stance models appropriate validation of emotion and a dialectical outlook, whereby it is acknowledged that two seemingly contradictory accounts can both be true (e.g. ‘I am an okay person and I can adapt my behaviours in this context to get a better outcome for myself and others’.

The full DBT programme includes group skills training, individual therapy sessions, skills coaching outside of sessions, structuring the environment and group consultation between therapists; however, partial versions of DBT, often called DBT-informed interventions, have been developed. DBT, including DBT-informed interventions, has been tested for a range of patient groups, including people without a diagnosis of personality disorder, where emotional dysregulation plays a role in the presenting issues [21–23]. It has found to be effective in reducing substance use and self-harm (both being behaviours that are often used by individuals to cope with intense, seemingly unmanageable affect) and problematic anger. A small number of case series and feasibility studies have explored DBT-informed interventions for people with bipolar disorder, with promising outcomes in terms of acceptability and likely clinical benefit; these vary in the extent to which they adhere to the traditional DBT programme versus include concepts and techniques influenced by DBT or other emotion regulation programmes [24]. In addition, except for one study [25], these studies do not focus specifically on inter-episode symptoms or include individuals with cyclothymic disorder.

With input from people with lived experience of bipolar disorder, and family members, we integrated concepts and techniques from DBT within a BA protocol used previously with individuals with bipolar depression [18]. An initial examination of the safety and acceptability of the resulting therapy was conducted within in a case series of 12 individuals with BD or CD and ongoing low mood or mood instability. At the time of writing, the acute treatment phase of the case series is complete and pre-determined rules to allow progression to the next phase of evaluation, a randomised controlled feasibility trial, have been met (no serious concerns about therapy safety; instances of reliable improvement exceed instances of reliable deterioration). The protocol for this feasibility trial is presented here.

The overall aim of this study is to determine the likely feasibility, acceptability and safety of the research procedures, including the therapy protocol, to design a future definitive randomised trial to determine the clinical and cost effectiveness of the STABILISE therapy programme.

## Objectives

(i) To inform the recruitment and timeline of a future fully-powered trial, by informing estimates of the number of participants who will need to be identified, approached, consented, randomised and who are likely to complete outcome assessments.
(ii) To refine future trial procedures by establishing the acceptability and experience of the trial process to participants. This includes acceptability and experience of randomisation and outcome measures.
(iii) To inform selection of the optimal primary outcome measure in a future trial by assessing the performance of selected candidate primary outcome measures with respect to level of acceptability to participants and participant-perceived relevance and value. Although not powered to detect a significant between group mean difference, we will scrutinise between group mean differences for each outcome measure, as well as relative rates of reliable improvement and deterioration, to assess sensitivity of the measure to the impact of the intervention.
(iv) To inform estimation of sample size for a future trial by measuring data completeness at follow up (participant attrition) and standard deviation of the likely primary outcome measure (to compare to reports in published literature).
(v) To characterise the comparator condition, treatment as usual, across individuals and sites.
(vi) To further explore the safety and acceptability of the treatment and, based on input from trial participants and clinicians, to further refine and develop the treatment manual and the procedures for training, supervising and assessing the competence of trial therapists.
(vii) To demonstrate feasibility outside of the lead site by including a second site.
(viii) To estimate the cost of the intervention.
(ix) To assess the feasibility of collecting health care resource utilisation data and health-related quality of life data using the EQ-5D-5L [26].
(x) To explore the feasibility of collecting momentary assessment (experience sampling) data at three time points (5 times per day, for 10 days, at each timepoint): the ultimate purpose of collecting these data in a future larger trial is to estimate mood instability and to examine process of change.

The criteria for progression to definitive trial are given in Table 1, presented in accordance with the framework proposed by Avery and colleagues [27].

**Table 1:** Criteria for progression to a definitive trial

	Red		Amber		Green	
Overall safety of intervention and trial procedures as determined by TSC and sponsor^a^	Cannot be made sufficiently safe for use in a definitive trial		Can be made sufficiently safe following modification		Sufficiently safe for use in a definitive trial	
Mean recruitment rate per site over 15 months	< 2 participants per month at both sites		< 2 participants per month at one site and target sample size not reached, with clear plan for improvement		≥ 2 participants at both sites OR < 2 participants at one site but overall target sample size reached^b^	
Outcome measure completion at 30-week follow-up point (completion of candidate primary outcome measure at primary end point)	< 55% of participants		Between ≥ 55 and < 75% of participants, with clear plan for improvement		≥ 75% of participants	
Level of completion of intervention (defined as attending at least 6 therapy sessions)^c^	< 50% of participants		50–89% of participants, with clear plan for improvement		≥ 90% of participants	

Red: Do not progress to the main trial (unless TSC agree exceptional circumstances and a mitigation plan is present); Amber: Progress if action plan to mitigate problems can be determined and agreed with the Trial Steering Committee (TSC); Green: Progress directly to the main trial

^a^Subsequent to reviewing information on adverse and serious adverse events arising during the trial

^b^To allow for a situation in which recruitment systems require a lead-in period at a site before becoming optimized, and this optimization period can then be accounted for in planning for a definitive trial

^c^Information will be collected on reasons for discontinuation to inform plan for enhancing retention in intervention and/or refinement of the estimand for a definitive trial [28]

## Method

This feasibility study protocol is reported according to the SPIRIT 2013 statement.

### Design

This feasibility study has a two-arm randomised parallel controlled trial design. Participants will be randomised on a 1:1 ratio to Treatment as Usual (TAU) only (control arm) or TAU plus STABILISE programme (intervention arm). Outcome measures will be completed at baseline and at three follow-up points: 14, 30 and 52 weeks post study entry. We have selected 30 weeks as the primary end point to allow this to be immediately following the intervention period. The study takes a mixed-method approach whereby evaluation of therapy acceptability and potential impact is explored both qualitatively and quantitatively.

### Setting and participants

The trial will be conducted across two sites in the U.K. (an NHS mental health Trust, and a specialist NHS outpatient psychological therapies service based at the University of Exeter) with participants allowed to be recruited from primary care services, secondary care mental health services and via self-referral. For details of study sites please see: https://www.isrctn.com/ISRCTN18207465.

Eligible participants will be aged 18 or over with a diagnosis of Bipolar Disorder (I, II, Other Specified Bipolar Disorder) or Cyclothymic Disorder, according to DSM-V [29] criteria assessed using the Structured Clinical Interview for DSM-V (SCID-5 [30]). Participants must report either current bipolar mood instability, defined as a score of at least 1.3 on the depression-elation subscale of the short form Affective Lability Scale (ALS [31]), or presence of at least mild depressive symptoms, defined as a score of at least 5 on the Patient Health Questionnaire (PHQ-9 [32]: see Additional File 1 for details of measures used). They must also be willing to engage in psychological therapy that focuses primarily on psychological work addressing ongoing bipolar symptoms or their impact on functioning, and must be able to attend regular therapy sessions. Participants must have sufficient competency in English such that study measures can be completed without the need for translation (to preserve the measure properties), have completed the intake measures, and be registered with a General Practice in the study site catchment area.

Participants will not be included if they are currently experiencing a manic episode (assessed using the SCID-5) or severe major depressive episode (assessed using the SCID-5; the Hamilton Depression Rating Scale [33], an established, validated measure of current depression symptoms, will be used with participants who meet criteria for a current depressive episode to establish severity). We will not include participants with current substance dependence according to The International Classification of Diseases, 11th revision (ICD-11 [34]) criteria; ICD-11 rather than DSM-V criteria are used to permit assessment of substance dependence rather than the broader construct of substance use disorder. Participants who present a risk of harm to themselves or others that cannot be safely managed in a community outpatient setting, will not be included. Receiving multiple, separate psychological interventions for BD simultaneously is not generally considered clinically advisable, therefore participants will not be eligible for the study if currently receiving a psychological intervention for BD at the point of study intake.

Medication status will not serve as an exclusion criterion but will be recorded. In both conditions participants will continue to be able to access routine care.

### Sample size

A total of 60 individuals will be recruited (target *n* = 30 in Devon, and *n* = 30 in Avon & Wiltshire). A sample size of 60 participants allows estimation of loss to follow-up within ± 12 percentage points, assuming 20% attrition, and is sufficient to estimate the standard deviation in the candidate primary outcome measures [35, 36].

### Randomisation, concealment of allocation and blinding

Participants eligible for the study will be randomised on a 1:1 ratio (with minimisation by trial site and medication status [currently prescribed medication for depression or Bipolar Disorder versus not prescribed such medication]) to Treatment as Usual (TAU) [control arm] or Treatment as Usual plus STABILISE programme (TAU + STABILISE) [intervention arm]. Allocation concealment during the randomisation process will be achieved through use of a validated password protected online randomisation tool programmed and hosted by the Exeter Clinical Trials Unit. The first six participants will be allocated using simple randomisation and then the minimisation procedure will commence, maintaining a stochastic element to the algorithm to allow concealment to be maintained. The minimisation algorithm will take account of the allocations of the first six participants. Participants will be enrolled into the randomisation system and informed of their allocation by an unblinded researcher.

Given the nature of the intervention, blinding of participants to allocation is not possible. Follow-up assessments will be conducted by separate researchers who are unaware of the participant’s allocation. At follow-up, outcome assessors will be instructed to maintain blinding by reminding participants not to reveal their allocation during the contact. Outcome assessors will be asked to indicate at follow-up which treatment they believe the participants received, allowing us to test blinding status and analyse any correlation with outcome. The outcome assessors’ blind will be maintained as far as possible; they will be deliberately unblinded only in exceptional circumstances where knowledge of the treatment arm is deemed essential to their management of the participant (e.g. certain Serious Adverse Events). Instances of deliberate or accidental un-blinding will be recorded; future data collection from that participant will be carried out by a member of the team who remains blinded. An unblinded researcher will collect data from participants that pertain to their allocation (qualitative and quantitative feedback about their experience of the study and of the therapy). All other members of the study team will be unblinded other than those performing statistical analyses on the data (health economist and statistician). Statistical and economic analyses will be performed by a statistician and health economists who are blinded to participant allocations.

### Recruitment

Participants will be identified from local NHS services including but not limited to primary care psychological therapies services, General Practices, secondary care mental health teams, early intervention services and secondary care psychological therapies services. In addition, advertisements for the study will be placed in healthcare settings, public places and distributed through traditional and social media, and third sector organisations. Potential participants who are interested in the study will be given the choice of contacting the researchers directly by telephone, email or post or completing a ‘permission to contact’ form which can be passed to the research team by their clinician or sent directly to the study team by the participant.

After a potential participant contacts or is referred to the study, a member of the research team will answer any initial questions and have an initial discussion about the study if the person wishes. Following receipt from the participant of a completed form gaining their consent for contact and an initial telephone screening call (‘permission to contact’ form), the researcher will arrange a time to complete this with the participant. In the screening call, the researcher will go through the inclusion and exclusion criteria with the participant, to give an indication of the likelihood that the participant will be eligible. Those likely to be eligible and willing to continue will be invited to the intake appointment.

At the appointment, the researchers will take written consent if the participant wishes to proceed and conduct the eligibility assessment. If the potential participant is eligible to partake in the study, is fully informed and has consented to participate, then they will be enrolled into the study.

Participants will be offered an honorarium of £20 for their participation at four points in the study: the intake assessment and the 14, 30 and 52 week follow-up points.

### Trial intervention

Using an approach informed by experience-based co-design [37], the STABILISE therapy programme was developed and refined through a series of workshops and other communications with patients and supporters, as well as input from therapists familiar with the component therapies.

Established psychological therapies for people with BD, such as CBT and group psychoeducation, include multiple skills to support people in managing symptoms and relapse, and some of these skills are applied to subsyndromal symptoms. STABILISE draws upon many of these skills. As with standard CBT, STABILISE includes a behavioural approach that seeks to promote re-engagement with valued activities and meaningful routine. Specifically, STABILISE recognises that instability in behaviours and affect in IEBS are related, and aims to help participants find a balanced, sustainable pattern of activity that enables them to live well within their situation, to support them to change their situation where possible and needed, and to make changes to patterns of behaviour that lead to problems or distress. This typically entails reducing mood-driven behaviour and increasing behaviours guided by the person’s values, plans or goals. These principles apply equally to depressed versus hypomanic states.

Standard techniques for bipolar symptom management tend to be based on the assumption that mood fluctuations build up over days and weeks, culminating in a relatively stable major episode. Consequently, these techniques may not be optimal for managing more rapid changes in affect. STABILISE draws upon techniques from DBT and emotion regulation therapies for affect management, and incorporates these within its behavioural framework. Recognising that people with bipolar disorder, particularly those with IEBS, have often experienced many years of threatening, uncontrollable shifts in affect, energy and motivation, STABILISE draws upon several key principles of DBT including an emphasis on validation of emotional experiences and a dialectical stance, balancing acceptance and change. For a summary of similarities and differences between STABILISE and established, related approaches please see Additional File 2.

The intervention consists of up to 20 individual therapy sessions (plus up to 2 initial assessment sessions) of behavioural therapy, delivered up to 7 months (30 weeks). Therapy ends after 20 sessions or 7 months, whichever is sooner, and participants can choose to space sessions out over more than a week if they wish or to take a short break from therapy. Sessions are an hour long as default but with the option for participants to agree shorter or longer (up to 75 min) session duration if needed. This is followed by a period of consolidation whereby participants can opt to see the therapist up to 3 times up until 12 months after starting therapy. All sessions will be audio-recorded (where participants consent to this) to allow for supervision and the refinement of a therapy adherence and competence measure.

Therapy will be delivered by at least three therapists with an existing training in cognitive behavioural, behavioural or dialectical behavioural therapy and experience of working with people with mood disorders. Therapists new to delivering the intervention will receive a 3-day training programme and all therapists will receive supervision every 1–2 weeks from the therapy developer or a STABILISE senior therapist whilst delivering therapy.

Therapy will be delivered face-to-face, online or by phone according to participant preference and what is feasible; local NHS service protocols will be followed in terms of telephone and online platform use. Face-to-face therapy will by default be delivered in the treatment centre; however, in keeping with the ethos of the approach (patient-centred, flexible, contextual and experiential) therapy sessions may take place outside of the centre if the participant wishes and it is appropriate and safe to do so. This may include practising activities together (e.g. visiting a shop) or sessions within the person’s home. Participants will complete the Beck Depression Inventory (BDI) [38] and Altman Self-Rating Mania Scale (ASRM) [39] prior to each session and the scores used to inform their care, as well as forming part of the research data collected.

It is anticipated that changes to the therapy protocol during the feasibility trial will be minor (i.e. will concern including or excluding specific techniques, or the wording and presentation of therapy materials, rather than the underpinning principles, therapeutic approach and overall structure). Changes to the therapy protocol will be timestamped allowing identification of which participants received which version.

The comparison arm in this trial is TAU. This was chosen because of the absence of a ‘gold standard’ therapy for inter-episode symptoms against which the novel therapy can be benchmarked. To characterise the content of TAU across trial sites, we will collect data on health services used by participants. There will be no restriction on the content of TAU; this includes no restrictions on participants accessing psychological therapies for bipolar disorder once entered into the study.

### Outcomes and measures

As this is a feasibility trial, the primary outcomes relate to the key feasibility objectives. Additional File 3 details how each feasibility objective will be assessed.

#### Demographic information

At the baseline eligibility assessment, demographic information (age, gender, ethnicity, perceived financial status, sexual orientation) will be collected.

#### Patient-reported clinical outcome measures

In addition to the PHQ-9 and ALS, the following clinical outcome measures will be completed at baseline, 14, 30 and 52 week follow-up (see Additional File 2 for measure details): Bech Rafaelsen Mania Scale (BRMS [40], Brief Quality of life in Bipolar Disorder scale (QoLBD [41], Generalised Anxiety Disorder Questionnaire (GAD-7 [42]), Bipolar Recovery Questionnaire (BRQ [43]), Life chart self-report (a self-report of number and duration of depressive and manic episodes in the period since last assessment, completed at 14, 30 and 52 week follow-up points only, with the support of the researcher; Health Economics Questionnaire (HEQ [44]), EQ-5D-5L (health related quality of life). At this stage, candidate future primary outcome measures include the PHQ-9, ALS, QoL.BD and BRQ.

#### Quantitative process measures

The theoretical model underpinning the therapy hypothesises that mood-related difficulties are maintained, at least in part, by individuals responding impulsively to intense mood states, and some of these responses further affecting mood as well as contributing to difficulties in everyday life. In order to test this hypothesis in a future, larger trial we plan to use the following self-report measures, which are included in the current study, to assess the acceptability of doing so: Positive and Negative Urgency Scales (PU & NU [45]),; Behavioural Activation in Depression Scale–Short Form (BADS-SF [46]).

As another means of assessing the process of change within therapy as part of a large, future trial, we plan to measure mood and activity repeatedly to allow us to look at the inter-relationships between these. In the current study, we plan to gather information on the feasibility and acceptability of collecting these data, having initially tested this method within our case series. Participants will be invited to report on their current mood and activity 5 times per day for 10 days via a purpose-built web application (momentary assessment block). These momentary assessment blocks will take place on three occasions: for 10 days following the intake assessment, for 10 days at 14 weeks post randomisation and for 10 days following the 30-week follow-up point.

#### Additional measures

At the 30-week follow-up point, participants will complete a feedback questionnaire within which they will be asked to rate (on a scale from 1 to 4) their satisfaction with the therapy, how acceptable it was and the likelihood they would recommend it to a friend (for those in the intervention arm), and their overall satisfaction with the research element of the therapy (both arms), and also to give written comment on their answers to each questions and any general comments on the therapy or research. A brief feedback questionnaire will be sent to those exiting the study early, at the point they exit, if they have indicated they would be willing to complete this.

To monitor and evaluate intervention and study safety, an ‘Asking about adverse events’ form will be completed following the intake assessment (a researcher will check on participants’ wellbeing within a week of the intake assessment), and at the 14-, 30- and 52-week follow-up points. Members of the research team will also follow up with participants in relation to any potential adverse events mentioned in ad-hoc communications, therapy contacts or via HEQ completion.

Participants will be invited to rank outcome measures according to which they would most wish to experience change upon. Rankings will be conducted at baseline, and at the 30-week follow-up point.

Participants will be asked about their concordance with their prescribed psychiatric medication using the single, overall compliance question from the Brief Adherence Rating Scale (BARS [47]) at intake, 14 and 30 weeks.

#### Qualitative interview

At 30 weeks (patient participants) and at the end of the trial (therapists), there will be audio-recorded qualitative interviews of approximately 60 min conducted by one of the research team exploring experiences of the therapy. Fifteen participants in the intervention arm, purposively sampled to span diversity in terms of demographic characteristics and outcome in terms of therapy attendance, will be interviewed. All therapists who have delivered at least one therapy session as part of the study will be invited to be interviewed.

The interview will allow participants/therapists to describe their views in detail. The interview will be informed by the topic areas highlighted in process evaluation guidance [48], namely fidelity and quality of intervention implementation, mechanisms of change and impact of context. Specifically, it will ask about how participants/therapists found the therapy, any perceived effects of the therapy, aspects that were helpful/unhelpful, and views on the length and delivery format of the treatment and the feasibility/acceptability of both the intervention and the outcome measurement (for participants). It will also ask about experienced process of change, including questions on hypothesised mechanisms but also leaving space for these to arise inductively from reports, as well as participants’ experiences of the impact of the context in which they received the intervention (their personal/social context, and the healthcare context). The interviews will follow a topic guide, but with flexibility to adapt this based on the answers given.

Data collection forms will be available on request from the Chief Investigator following completion of the trial.

### Procedure

**Fig. 1:**
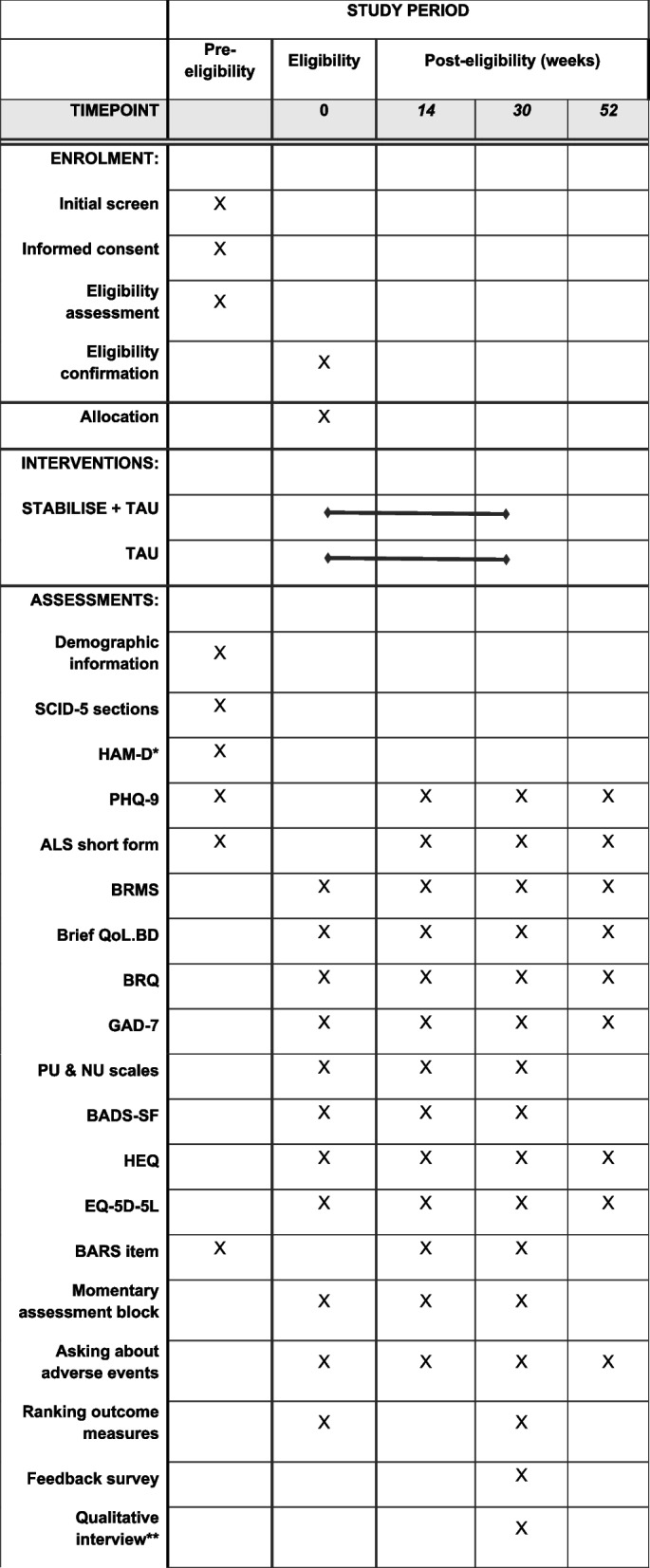
Schedule of enrolment, interventions, and assessments (following SPIRIT template)

Informed consent will be taken prior to or at commencement of the eligibility assessment by a member of the study team; consent will be re-affirmed verbally prior to qualitative interviews. Following the initial eligibility assessment and completion of baseline measures, participants will be randomised to either STABILISE plus TAU, or TAU. Those allocated to the intervention arm will commence treatment approximately 2 weeks after being deemed eligible. Assessments at 14, 30 and 52 weeks will involve participants completing self-report measures either online or on paper, according to their preference, as well as speaking to a member of the research team (online, by telephone or in person) to complete the measures that require contemporaneous researcher involvement. A default procedure will be followed in cases where participants do not respond to requests to complete measures, with the procedure being tailored in accordance with individual patient preferences, as discussed following acceptance to the study. Figure 1 displays the measures completed at each time point.

## Data analysis

### Statistical analyses

Given the feasibility design, data analysis will focus on a descriptive analysis of the feasibility objectives of the study. We will report recruitment rates (average number of participants recruited per month) overall and by site. Retention and completion of outcome measures at follow-up timepoints will be reported as numbers and percentages, overall and by each treatment group. Participant characteristics at baseline will be reported descriptively for each treatment group, using mean (SD) for continuous variables, and percentages for categorical variables.

Outcome measures will be reported descriptively for each treatment group at baseline and at each post-randomisation follow-up, i.e. 14, 30 and 52 weeks. For 30- and 52-week follow-up data, the between group mean differences for continuous variables (intervention minus control group) will be reported with a 95% CI. The between group mean difference will be derived from a linear regression model, which will include treatment group as a fixed effect and will also adjust for study site and use of medication for BDs (factors used in the randomisation procedure). The feedback questionnaire will be reported descriptively for those items that relate to the STABILISE + TAU group only; the overall satisfaction item will be reported descriptively by treatment group and with a between group mean difference and 95% CI (using a linear regression model as for the clinical outcomes).

Given the feasibility nature of the trial, all participants will be analysed according to their randomised group irrespective of treatment actually received (intention to treat principle). Analyses will include observed data only; no imputation of missing outcome data will be performed. To explore potential mechanisms of missingness, we will report the mean (SD) for baseline PHQ-9 and GAD-7 for participants with and without completion of all four potential primary clinical outcome measures at 14 weeks, overall and by treatment group.

Serious adverse events (SAEs) will be reported as number of SAEs using the ‘as treated’ approach: participants in the STABILISE + TAU group will be classed as TAU only if they do not attend at least one therapy session. (It is assumed that participants allocated to TAU only will not be able to access therapy sessions.) SAEs will be reported numerically by ‘as treated’ group during the 52-week trial period, and as the number and percentage of individual participants experiencing one or more SAEs during the 52-week trial period. In addition, a risk difference and odds ratio for experiencing one or more SAEs during the 52-week trial period will be reported, with 95% CIs. Analyses will be performed using Stata v18 or later, by a statistician who is blinded to group allocation.

### Economic analyses

The economic analyses will follow the same approach as the statistical analysis. We will use an intention-to-treat analysis, taking the NHS and social care perspective. As this is a feasibility trial, we will report descriptive data only for each trial arm, with confidence intervals for between group comparisons. No *p* values will be reported. Primary analysis will be based on complete case data. However, losses to follow-up at data collection points will be presented for both primary and secondary outcomes. Missing outcome data will also be discussed, along with its causes.

In order to cost the intervention, we will collect information on the resource use and costs of delivering the STABILISE intervention depending the type of delivery, i.e. face-to-face, online or by phone. It will include costs on training and salaries, course materials, travel expenses, supervision and administration costs. The costs of the intervention will be estimated as a cost per participant. Sensitivity analyses will be conducted using the minimum and maximum number of sessions of the STABILISE intervention.

### Qualitative data analysis

Qualitative interviews will be audio-recorded and then transcribed. Participants’ responses will be read closely and coded using a framework approach, informed by the framework for assessing intervention acceptability proposed by Sekhon and colleagues [49]. Data relevant to each category will be compared and summarised and non-conforming cases examined closely to understand similarities and differences in perspective. Interview recordings will be transcribed by a member of the study team, or a suitable individual working with the team who has signed a confidentiality agreement.

## Data management and storage

The General Data Protection Regulation [50] will be followed. Study data will be held in linked anonymous form. Participants will be identified by a code, with the document linking participant name with ID code being stored separately to the rest of the data, and accessible only to measures of the research team. Personal data will be transferred and stored only where necessary. Details of the storage plan for each data format are listed in Additional File 4.

The project CI will act as custodian for the data. If the CI leaves employment of the University of Exeter, a data custodian will be appointed by the University of Exeter (a member of the research group or of Exeter IT Services) who will manage continued archiving of the data. Anonymised data will be retained for 20 years. Personally identifiable information will be stored for 12 months after the end of the study to enable sufficient time for the study data to be analysed and a summary of the results to be sent to participants who requested these at study entry.

The study team members will have access to the final dataset, with the agreement of the CI.

Consent will be sought from participants to share anonymised data with other researchers for secondary analyses. Because of the potentially sensitive nature of the data and the potential for participants to be identifiable by their data by some members of the public, the public will not be given unrestricted access to the data. In accordance with good practice and institutional policy the research database will be registered with the University of Exeter public access database. The dataset will be anonymous and will be registered with a metadata only record, allowing the research team to control access to the dataset, restricting it to appropriately qualified third parties.

## Anticipated risks and benefits

The administration of a novel therapeutic intervention raises the potential of risk to those in the trial, as well as potential benefit. We believe that the intervention is unlikely to pose significant risks: the approach is an adaptation of two widely used existing therapies (Behavioural Activation and Dialectical Behavioural Therapy), both of which have been delivered previously by our team to people with bipolar disorder in case series and trial contexts with no significant concerns about therapy safety resulting [18, 25]. We are currently completing a case series evaluation of the STABILISE approach: at the time of writing all 12 participants have completed the acute phase of therapy with no significant concerns arising regarding the safety of the intervention. Reactions to therapy will be monitored by therapists and within clinical supervision and standard operating procedures relating to monitoring and reporting adverse events will be followed.

In this study, participants are allocated at random to receive either TAU, or TAU plus the novel intervention. Therefore, there is a risk that a participant may be disappointed by allocation to the former arm. The inclusion of a treatment as usual comparator arm is necessary in order to address some of the feasibility aims (to establish the acceptability of procedures to be used in a definitive trial, including randomisation). Participants will be informed of the nature and purpose of the randomisation element. They will be called by an unblinded member of the study team to inform them of their allocation; their response to this will be monitored including the offer of a follow-up call the following day. Participants in both arms will be directed towards sources of support in the local community and nationally. It will be made clear to those in the usual care arm that whilst participants currently receiving psychological therapy for bipolar disorder will not be eligible to join the study, no restrictions are placed on the treatments that participants can choose to access outside of the study whilst they are a part of it. Because the treatment is experimental, it is not currently planned that it will be routinely offered to trial participants following study completion.

There is a chance that completion of study measures may induce distress. Our experience of using similar methods in previous research projects is that a significant adverse reaction is rare and if it occurs it is transient. The information sheet will make clear to participants that filling in the measures may temporarily lower mood or cause distress. The therapist/researcher will ask participants if they suffer any adverse reaction completing measures and will follow service or research distress management protocols if a significant level of distress is observed. Participants will be contacted within a week of completing the initial assessment to check on their welfare and to develop a bespoke plan for supporting participants as they move through the study (e.g. texts to check in with them, occasional planned calls, etc.).

The sponsor holds indemnity policies to allow compensation of trial participants if formal claims of harm are made and upheld.

### Adverse events

We will monitor and record both serious and non-serious adverse events. Adverse event reports will be solicited at follow-up points and monitored during all research contacts and through inspection of HEQ responses. An adverse event (AE) is defined by the HRA as any untoward occurrence or worsening of an unintended sign, symptoms or disease that occurs during participation, regardless of any relation to participation. In the STABILISE study, an adverse event will be recorded only when medical/clinical assessment or treatment is sought for a new or worsening sign, symptom or disease, unless the event appears to be a result of participation in the study. An adverse event will be classified as serious if it results in death, life threatening injury or condition, permanent impairment to a body structure, body function or the capacity of the individual to function in daily life, hospitalisation or extension of existing hospitalisation, foetal death or a congenital abnormality or birth defect.

Symptoms of bipolar disorder themselves are not defined as adverse events. However, deterioration in mental state that results in the person accessing emergency support will be classed as an adverse event.

Any AEs that researchers or therapists believe may be study related, may merit a classification of ‘severe’ (even if not technically meeting SAE criteria) or may constitute an SAE will be required to be reported to the site PI, Trial Manager and the CI within 24 h. Other AEs must be reported to the PI, Trial Manager and CI within 1 week. The reporting period for all events and reactions will be from intake assessment to completion of the 52 week follow-up point.

The only expected reactions to the therapy and/or trial procedures are distress or symptom exacerbation (including behaviours such as self-harm, suicidal behaviour or substance use which may be considered to be part of the symptom set that individual typically experiences or may be considered to be coping responses in the face of distress or symptom exacerbation).

It is recognised that those participants in the intervention arm have more contact with the study team (and therefore more opportunity to report adverse events) than do those in the usual care arm, by virtue of attending therapy sessions. Thus, we may expect a higher rate of adverse events reported in the former arm. In this study particular attention will be paid to those events that appear to be caused by research or therapy participation.

### Criteria for discontinuation

Individual participants will discontinue their involvement if either (i) the participant does not wish to continue with the intervention and study; (ii) the participant experiences an unexpected serious adverse reaction (mental or physical health event that results in significant impairment, hospitalisation or death) that is judged to be the direct result of the intervention or trial participation, where discontinuation is judged to be in the best interests of the participant by the participant or sponsor (with advice from the Trial Steering Committee); (iii) the participant and/or therapy/research team believe that the intervention or trial participation will result in, or is likely to result in, a serious adverse reaction if continued; or (iv) the participant does not attend more than three consecutive therapy sessions without explanation; this will be judged to indicate discontinuation of therapy. Over the period that the participant does not attend sessions, efforts will be made by the therapist to contact the participant. Participant choice to discontinue therapy will not be assumed to mean automatic withdrawal from the trial.

Should an unexpected serious adverse reaction occur to either the therapy or the trial procedures, and this is judged to be directly related to trial participation or to the therapy, the trial will be temporarily halted pending investigation and analysis of the extent to which future risk can be mitigated against. If it is judged that this is not possible, the trial will be discontinued. This process will be led by the sponsor in collaboration with the TSC chair and CI. Should information come to light from external sources that indicate that the therapy intervention or trial procedures are unsafe, the same process will be followed.

## Patient and public involvement

Throughout this study, we are working with a patient and public involvement panel (PPI panel) of individuals with personal experience of bipolar disorder, or who have a relative with bipolar disorder. The panel have contributed to the design of the therapy protocol and the materials for patients such as the information sheet, consent form and interview topic guide; a subgroup contributed to the initial design of the study itself. The panel will advise on the running of this study throughout.

## Trial governance

The Trial Steering Committee (TSC) includes independent members with academic and/or clinical expertise, and/or expertise based on personal experience. Also included are the CI and trial statistician. The TSC will meet approximately five times over the life of the project and will be responsible for advising the trial team on the conduct of the study. Given the relatively small scale of the trial, a separate Data Monitoring Committee (DMC) has not been convened. Instead, the DMC functions (reviewing data collected including adverse events and making recommendations for the future conduct of the trial) are included within the terms of reference of the TSC.

Approval will be sought from the sponsor and the relevant national regulatory bodies for all substantive changes to the protocol and these will be communicated externally via an update to the ISRCTN registration record.

### Role of the funder and sponsor

Ultimate authority over the management of the study lies with the study sponsor. Neither the funder nor the sponsor of the study were involved in the design of the study and will not be involved in the collection, analysis or interpretation of data, or the writing of the study report.

### Study approvals

The conduct of the trial will be in accordance with the Helsinki Declaration. The study has received approval from the U.K. Health Research Authority Research Ethics Service (IRAS ID: 335983), and from all relevant local approval bodies. 

## Dissemination

The data arising from the study will be the property of the University of Exeter and the Chief Investigator. On completion of the study, the data will be analysed and tabulated and a Final Study Report prepared. The full study report will be made available in open access form via the University of Exeter and publication via a peer reviewed journal will be sought, acknowledging contributions to the study in line with journal policy. Findings will also be disseminated through academic conference presentations or posters, and publicly following a dissemination strategy to be developed with the PPI panel. Participants will be asked to state whether they wish to receive a copy of the findings which they will be sent once these have been finalised. In order to protect participant anonymity, the raw data will not be made publicly accessible.

Authorship of the final trial report will be in line with the recommendations of the International Committee of Medical Journal Editors [51].

## Trial Status

The start date of the trial was 28th March 2024. Recruitment is running from April 2024 to June 2025 inclusive. Follow up will last 52 weeks, with the entire study period lasting for 28 months.

## Discussion

### Expected findings

This trial is designed to assess the feasibility, acceptability and safety of a randomised controlled trial of an adapted form of behavioural therapy for individuals with ongoing bipolar symptoms or cyclothymic disorder. To do so, we will employ a mixed-methods approach whereby information about intervention acceptability is derived both from quantitative measures of attendance and completion and participant acceptability ratings, as well as from qualitative information gathered via interview and feedback questionnaire. Our study builds on an intervention co-development phase with individuals with lived experience of bipolar disorder, and also upon a case series in which 12 people received the resulting intervention. The case series met pre-determined continuation criteria, allowing progression to the current trial to address uncertainties prior to conducting a definitive, randomised, controlled trial of the intervention. Progression to the definitive trial will be determined according to performance against the set of continuation criteria described here.

### Limitations

As this is a feasibility trial, it is not designed to assess the clinical effectiveness of the intervention. Whilst this study will benefit from the information provided by the inclusion of a second trial site, the feasibility conclusions drawn may not apply to all possible additional sites in a future definitive trial. Consequently, planning and decision-making with respect to any future trial will take into account additional information available, outside of the scope of the current study.

## Conclusion

This study is an early step in establishing the clinical and cost effectiveness of an adapted form of behavioural therapy for people who find their bipolar mood issues persist outside of major affective episodes. In the longer term, if effectiveness is demonstrated, the intention is that it could be routinely delivered within the U.K. health service.

## Supplementary Information

Additional File 1. Details of measures used. Title, construct addressed and scale and item details for measures used in the trial.

Additional File 2. Relationship between STABILISE and related psychological interventions for people with bipolar disorder. Table listing conceptual and technical similarities and differences between STABILISE and related interventions that have been adapted or developed for use with people with bipolar disorder.

Additional File 3. Means of measuring progress against study objectives. Study objectives with details of how progress or performance against each objective will be measured.

Additional File 4. Data storage details. Means of storing study data according to data type.

## Data Availability

Data sharing is not applicable to this article as no datasets were generated or analysed during the current study.

## Abbreviations

AE: Adverse event
ALS: Affective Lability Scale
ASRM: Altman Self-Rating Mania Scale
BA: Behavioural activation therapy
BADS-SF: Behavioral Avoidance in Depression Scale–Short Form
BARS: Brief Adherence Rating Scale
BD: Bipolar disorders
BDI: Beck depression inventory
BRQ: Bipolar Recovery Questionnaire
CI: Confidence interval
CI: Chief investigator
DBT: Dialectical behavior therapy\
DSM-V: Diagnostic and Statistical Manual of Mental Disorders, fifth edition
EQ-5D-5L: EuroQoL 5 Dimension 5 Level
GAD-7: Generalised Anxiety Disorder 7-item scale
HAM-D: Hamilton Depression Rating Scale
HEQ: Health Economics Questionnaire
ICD-11: International Classification of Diseases 11th Revision
IEBS: Inter-episode bipolar symptoms
NHS: U.K. National Health Service
NIHR: National Institute for Health Research
PHQ-9: Patient Health Questionnaire 9 item scale
PPI: Patient and public involvement
QALYS: Quality adjusted life years
QoL-BD: Quality of Life in Bipolar Disorder scale
SAE: Serious adverse event
SCID-5: Structured clinical interview for DSM-V
SD: Standard deviation
STABILISE: Experimental treatment evaluated in this study
TAU: Treatment as usual
TSC: Trial Steering Committee
PU & NU: Positive and Negative Urgency Scales

## References

[1] Pini S, De Queiroz V, Pagnin D, Pezawas L, Angst J, Cassano GB, Wittchen HU. Prevalence and burden of bipolar disorders in European countries. Eur Neuropsychopharmacol. 2005;15(4):425–34. 15935623 10.1016/j.euroneuro.2005.04.011

[2] Simon J, Pari AA, Wolstenholme J, Berger M, Goodwin GM, Geddes JR. The costs of bipolar disorder in the United Kingdom. Brain and Behavior. 2021;11(10). 34523820 10.1002/brb3.2351 PMC8553306

[3] Paykel ES, Abbott R, Morriss R, Hayhurst H, Scott J. Sub-syndromal and syndromal symptoms in the longitudinal course of bipolar disorder. Br J Psychiatry. 2006;189(2):118–23. 16880480 10.1192/bjp.bp.105.013870

[4] MacQueen GM, Marriott M, Begin H, Robb J, Joffe RT, Young LT. Subsyndromal symptoms assessed in longitudinal, prospective follow-up of a cohort of patients with bipolar disorder. Bipolar Disord. 2003;5(5):349–55. 14525555 10.1034/j.1399-5618.2003.00048.x

[5] Gershon A, Eidelman P. Inter-episode affective intensity and instability: predictors of depression and functional impairment in bipolar disorder. J Behav Ther Exp Psychiatry. 2015;1(46):14–8. 10.1016/j.jbtep.2014.07.005 PMC4254202 25164093

[6] Kochman FJ, Hantouche EG, Ferrari P, Lancrenon S, Bayart D, Akiskal HS. Cyclothymic temperament as a prospective predictor of bipolarity and suicidality in children and adolescents with major depressive disorder. J Affect Disord. 2005;85(1–2):181–9. 15780688 10.1016/j.jad.2003.09.009

[7] Samalin L, de Chazeron I, Vieta E, Bellivier F, Llorca PM. Residual symptoms and specific functional impairments in euthymic patients with bipolar disorder. Bipolar Disord. 2016;18(2):164–73. 26946486 10.1111/bdi.12376

[8] Roux P, Raust A, Cannavo AS, Aubin V, Aouizerate B, Azorin JM, Bellivier F, Belzeaux R, Bougerol T, Cussac I, Courtet P. Associations between residual depressive symptoms, cognition, and functioning in patients with euthymic bipolar disorder: results from the FACE-BD cohort. Br J Psychiatry. 2017;211(6):381–7. 29051175 10.1192/bjp.bp.117.201335

[9] Stanislaus S, Faurholt-Jepsen M, Vinberg M, Coello K, Kjaerstad HL, Melbye S, Sletved KS, Christensen EM, Frost M, Bardram JE, Kessing LV. Mood instability in patients with newly diagnosed bipolar disorder, unaffected relatives, and healthy control individuals measured daily using smartphones. J Affect Disord. 2020;15(271):336–44. 10.1016/j.jad.2020.03.049 32479333

[10] Judd LL, Schettler PJ, Akiskal HS, Coryell W, Leon AC, Maser JD, Solomon DA. Residual symptom recovery from major affective episodes in bipolar disorders and rapid episode relapse/recurrence. Arch Gen Psychiatry. 2008;65(4):386–94. 18391127 10.1001/archpsyc.65.4.386

[11] Alda M, McKinnon M, Blagdon R, Garnham J, MacLellan S, O’Donovan C, Hajek T, Nair C, Dursun S, MacQueen G. Methylene blue treatment for residual symptoms of bipolar disorder: randomised crossover study. Br J Psychiatry. 2017;210(1):54–60. 27284082 10.1192/bjp.bp.115.173930

[12] Yilmaz S, Huguet A, Kisely S, Rao S, Wang J, Baur K, Price M, O’Mahen H, Wright K. Do psychological interventions reduce symptoms of depression for patients with bipolar I or II disorder? A meta-analysis Journal of affective disorders. 2022;15(301):193–204. 10.1016/j.jad.2021.12.112 35007645

[13] Mazzarini L. The treatment of cyclothymia. The treatment of bipolar disorder: integrative clinical strategies and future directions. 2017;9:123.

[14] Ciharova M, Furukawa TA, Efthimiou O, Karyotaki E, Miguel C, Noma H, Cipriani A, Riper H, Cuijpers P. Cognitive restructuring, behavioral activation and cognitive-behavioral therapy in the treatment of adult depression: a network meta-analysis. J Consult Clin Psychol. 2021;89(6):563. 34264703 10.1037/ccp0000654

[15] Cuijpers P, Noma H, Karyotaki E, Vinkers CH, Cipriani A, Furukawa TA. A network meta-analysis of the effects of psychotherapies, pharmacotherapies and their combination in the treatment of adult depression. World Psychiatry. 2020;19(1):92–107. 31922679 10.1002/wps.20701 PMC6953550

[16] Richards DA, Ekers D, McMillan D, Taylor RS, Byford S, Warren FC, Barrett B, Farrand PA, Gilbody S, Kuyken W, O’Mahen H. Cost and outcome of behavioural activation versus cognitive behavioural therapy for depression (COBRA): a randomised, controlled, non-inferiority trial. The Lancet. 2016;388(10047):871–80. 10.1016/S0140-6736(16)31140-0 PMC5007415 27461440

[17] Weinstock LM, Melvin C, Munroe MK, Miller IW. Adjunctive behavioral activation for the treatment of bipolar depression: a proof of concept trial. J Psychiatr Pract. 2016;22(2):149–58. 27138086 10.1097/PRA.0000000000000142 PMC4855692

[18] Wright K, Mostazir M, Bailey E, Dunn BD, O’Mahen H, Sibsey M, Thomas Z. Adapted Behavioural activation for bipolar depression: a randomised multiple baseline case series. Brain Sci. 2022;12(10):1407. 36291340 10.3390/brainsci12101407 PMC9599144

[19] Linehan M. Cognitive-behavioral treatment of borderline personality disorder. New York, NY: Guilford press; 1993.

[20] Swales MA, Dunkley C. Structuring the wider environment and the DBT Team. The Oxford Handbook of Dialectical Behaviour Therapy. 2018;25:217.

[21] DeCou CR, Comtois KA, Landes SJ. Dialectical behavior therapy is effective for the treatment of suicidal behavior: A meta-analysis. Behav Ther. 2019;50(1):60–72. 30661567 10.1016/j.beth.2018.03.009

[22] Haktanır A, Callender KA. Meta-analysis of dialectical behavior therapy (DBT) for treating substance use. Research on education and psychology. 2020;4(Special Issue):74–87.

[23] Ciesinski NK, Sorgi-Wilson KM, Cheung JC, Chen EY, McCloskey MS. The effect of dialectical behavior therapy on anger and aggressive behavior: a systematic review with meta-analysis. Behav Res Ther. 2022;1(154): 104122. 10.1016/j.brat.2022.104122 35609374

[24] Jones BD, Umer M, Kittur ME, Finkelstein O, Xue S, Dimick MK, Ortiz A, Goldstein BI, Mulsant BH, Husain MI. A systematic review on the effectiveness of dialectical behavior therapy for improving mood symptoms in bipolar disorders. International Journal of Bipolar Disorders. 2023;11(1):6. 36739574 10.1186/s40345-023-00288-6 PMC9899872

[25] Wright K, Dodd AL, Warren FC, Medina-Lara A, Dunn B, Harvey J, Javaid M, Jones SH, Owens C, Taylor RS, Duncan D. Psychological therapy for mood instability within bipolar spectrum disorder: a randomised, controlled feasibility trial of a dialectical behaviour therapy-informed approach (the ThrIVe-B programme). International Journal of Bipolar Disorders. 2021;9:1–3. 34195864 10.1186/s40345-021-00226-4 PMC8245616

[26] Herdman M, Gudex C, Lloyd A, Janssen MF, Kind P, Parkin D, Bonsel G, Badia X. Development and preliminary testing of the new five-level version of EQ-5D (EQ-5D-5L). Qual Life Res. 2011;20:1727–36. 21479777 10.1007/s11136-011-9903-x PMC3220807

[27] Avery KN, Williamson PR, Gamble C, Francischetto EO, Metcalfe C, Davidson P, Williams H, Blazeby JM. Informing efficient randomised controlled trials: exploration of challenges in developing progression criteria for internal pilot studies. BMJ Open. 2017;7(2): e013537. 28213598 10.1136/bmjopen-2016-013537 PMC5318608

[28] Kahan BC, Hindley J, Edwards M, Cro S, Morris TP. The estimands framework: a primer on the ICH E9 (R1) addendum. BMJ. 2024;384. 10.1136/bmj-2023-076316 PMC10802140 38262663

[29] American Psychiatric Association. Diagnostic and Statistical Manual of Mental Disorders. 5th ed. 2013.

[30] First MB, Williams JB, Karg RS, Spitzer RL. Structured Clinical Interview for DSM-5 Disorders–Research Version (SCID-5-RV). Arlington: American Psychiatric Assocation. 2014.

[31] Oliver MN, Simons JS. The affective lability scales: development of a short-form measure. Personality Individ Differ. 2004;37(6):1279–88.

[32] Kroenke K, Spitzer RL, Williams JB. The PHQ-9: validity of a brief depression severity measure. J Gen Intern Med. 2001;16(9):606–13. 11556941 10.1046/j.1525-1497.2001.016009606.x PMC1495268

[33] Hamilton M. A rating scale for depression. J Neurol Neurosurg Psychiatry. 1960;23(1):56. 14399272 10.1136/jnnp.23.1.56 PMC495331

[34] World Health Organization. *ICD-11:* *International classification of diseases* (11th revision). 2022.

[35] Julious SA. Sample size of 12 per group rule of thumb for a pilot study. Pharm Stat J Appl Stat Pharm Ind. 2005;4(4):287–91.

[36] Whitehead AL, Julious SA, Cooper CL, Campbell MJ. Estimating the sample size for a pilot randomised trial to minimise the overall trial sample size for the external pilot and main trial for a continuous outcome variable. Stat Methods Med Res. 2016;25(3):1057–73. 26092476 10.1177/0962280215588241 PMC4876429

[37] Fylan B, Tomlinson J, Raynor DK, Silcock J. Using experience-based co-design with patients, carers and healthcare professionals to develop theory-based interventions for safer medicines use. Res Social Adm Pharm. 2021;17(12):2127–35. 34187746 10.1016/j.sapharm.2021.06.004

[38] Beck AT, Ward CH, Mendelson M, Mock J, Erbaugh J. An inventory for measuring depression. Arch Gen Psychiatry. 1961;4:561–71. 13688369 10.1001/archpsyc.1961.01710120031004

[39] Altman EG, Hedeker D, Peterson JL, Davis JM. The Altman self-rating mania scale. Biol Psychiat. 1997;42(10):948–55. 9359982 10.1016/S0006-3223(96)00548-3

[40] Bech P, Rafaelsen OJ, Kramp P, Bolwig TG. The mania rating scale: scale construction and inter-observer agreement. Neuropharmacology. 1978;17(6):430–1. 673161 10.1016/0028-3908(78)90022-9

[41] Michalak EE, Murray G, CREST. BD. Development of the QoL. BD: a disorder‐specific scale to assess quality of life in bipolar disorder. Bipolar Disord. 2010;12(7):727–40. 21040290 10.1111/j.1399-5618.2010.00865.x

[42] Spitzer RL, Kroenke K, Williams JB, Löwe B. A brief measure for assessing generalized anxiety disorder: the GAD-7. Arch Intern Med. 2006;166(10):1092–7. 16717171 10.1001/archinte.166.10.1092

[43] Jones S, Mulligan LD, Higginson S, Dunn G, Morrison AP. The bipolar recovery questionnaire: psychometric properties of a quantitative measure of recovery experiences in bipolar disorder. J Affect Disord. 2013;147(1):34–43. 23182591 10.1016/j.jad.2012.10.003

[44] Simon, J, Mayer, S. HEQ (Health Economics Questionnaire), Version 08–09–2016. Vienna: Department of Health Economics, Center for Public Health, Medical University of Vienna. 2016.

[45] Cyders MA, Smith GT, Spillane NS, Fischer S, Annus AM, Peterson C. Integration of impulsivity and positive mood to predict risky behavior: development and validation of a measure of positive urgency. Psychol Assess. 2007;19(1):107. 17371126 10.1037/1040-3590.19.1.107

[46] Manos RC, Kanter JW, Luo W. The behavioral activation for depression scale–short form: development and validation. Behav Ther. 2011;42(4):726–39. 22036000 10.1016/j.beth.2011.04.004

[47] Byerly MJ, Nakonezny PA, Rush AJ. The Brief Adherence Rating Scale (BARS) validated against electronic monitoring in assessing the antipsychotic medication adherence of outpatients with schizophrenia and schizoaffective disorder. Schizophr Res. 2008;100(1–3):60–9. 18255269 10.1016/j.schres.2007.12.470

[48] Skivington K, Matthews L, Simpson SA, Craig P, Baird J, Blazeby JM, Boyd KA, Craig N, French DP, McIntosh E, Petticrew M. A new framework for developing and evaluating complex interventions: update of Medical Research Council guidance. BMJ. 2021;374. 10.1136/bmj.n2061 PMC8482308 34593508

[49] Sekhon M, Cartwright M, Francis JJ. Acceptability of healthcare interventions: an overview of reviews and development of a theoretical framework. BMC Health Serv Res. 2017;17:1–3. 28126032 10.1186/s12913-017-2031-8 PMC5267473

[50] European Union. Regulation (EU) 2016/679 of the European Parliament and of the Council of 27 April 2016 on the protection of natural persons with regard to the processing of personal data and on the free movement of such data.

[51] International Committee of Medical Journal Editors. Defining the role of authors and contributors. Available from: https://www.icmje.org/recommendations/browse/roles-and-responsibilities/defining-the-role-of-authors-and-contributors. Cited 2024 June 19.

