# Supplementary material for: A study protocol for a randomized controlled feasibility trial of behavioural therapy for interepisode bipolar symptoms (STABILISE)
Source: Pilot Feasibility Stud. 2025 Jul 10;11:97. doi: 10.1186/s40814-025-01678-6 (PMC12243248; doi:10.1186/s40814-025-01678-6)
Supplement: Supplementary file 1 — Additional File 1. Details of measures used. Title, construct addressed and scale and item details for measures used in the trial. [file 40814_2025_1678_MOESM1_ESM.docx]

Additional File 1: Details of measures used

| Measure Title (Acronym) | Construct Addressed | Scale and Item Details |
| --- | --- | --- |
| Patient Health Questionnaire (PHQ-9) | Current depressive symptoms | Self report. Nine items rated on a scale from 0 (Not at all) to 3 (nearly every day) |
| Affective Lability Scale – short form (ALS) | Affective lability | Self report. Eighteen items rated on a scale from 0 (very undescriptive of me) to 3 (very descriptive of me). Comprises three subscales: depression-elation (8 items), anxiety-depression (5 items) and anger (5 items). |
| Structured Clinical Interview for DSM-V (SCID-5) | Research Diagnosis | Semi-structured interview. Sections used include: mood disorders, psychosis screener. |
| Beck Depression Inventory (BDI) | Current depressive symptoms | Self report. Twenty items rated on a scale from 0 to 3 (anchors unique to each item). |
| Altman Self-Rating Mania Scale (ASRM) | Current hypomanic / manic symptoms | Self report. Five items rated on a scale from 0 to 4 (anchors unique to each item). |
| Bech-Rafaelsen Mania Scale (BRMS) | Current hypomanic / manic symptoms | Observer rated. Eleven items rated on a scale from 0 to 3 (anchors unique to each item). |
| Brief Quality of Life in Bipolar Disorder Scale (QoL.BD) | Quality of life | Self-report. Twelve items rated on a scale from 1 (strongly disagree) to 5 (strongly agree). |
| Generalised Anxiety Disorder (GAD-7) | Current anxiety symptoms | Self report. Seven items rated on a scale from 0 (Not at all) to 3 (nearly every day) |
| Bipolar Recovery Questionnaire (BRQ) | Sense of personal recovery | Self report. Thirty six items rated on a visual analogue scale from 0 (strongly disagree) to 100 (strongly agree). |
| Positive Urgency and Negative Urgency scales (PU & NU) | Impulsive responding to positive mood (PU); to negative mood (NU) | Self report. Each scale contains 4 items rated on a scale from 1 (agree strongly) to 4 (disagree strongly). |
| Behavioural Activation in Depression Scale – Short Form (BADS-SF) | Behavioural approach versus avoidance | Self report. Nine items rated from 0 (not at all) to 6 (completely). |
| Satisfaction with therapy items [bespoke to this study] | Satisfaction with the therapy / study | Self report. Four items rated on a scale from 1 to 4 (anchors unique to each item). Three items concern satisfaction with therapy (completed only by those in STABILISE+TAU arm) and one concerns satisfaction with the research (completed by all participants). |
| Brief Adherence Rating Scale (BARS) | Concordance with medication prescribed | Self report. Participant estimates the % of times (0-100) taken medication as prescribed over given time period. |
| Health Economic Questionnaire (HEQ) | Health economic information including use of health services | Self report. Number of questions expand or contract based upon previous answers. |
| EuroQol 5 Dimensions 5 Levels (EQ-5D-5L) | Health related quality of life | Self report. Five items rated from 0 (no problems) to 5 (extreme problems) |
