# Supplementary material for: A study protocol for a randomized controlled feasibility trial of behavioural therapy for interepisode bipolar symptoms (STABILISE)
Source: Pilot Feasibility Stud. 2025 Jul 10;11:97. doi: 10.1186/s40814-025-01678-6 (PMC12243248; doi:10.1186/s40814-025-01678-6)
Supplement: Supplementary file 2 — Additional File 2. Relationship between STABILISE and related psychological interventions for people with bipolar disorder. Table listing conceptual and technical similarities and differences between STABILISE and related interventions that have been adapted or developed for use with people with bipolar disorder. [file 40814_2025_1678_MOESM2_ESM.docx]

Additional File 2: Relationship between STABILISE and related psychological interventions for people with bipolar disorder

| Intervention | Similarities | Differences |
| --- | --- | --- |
| IPSRT | Conceptual: notion that behaviour (routine and interpersonal interactions) contribute to exacerbations in mood difficulties.  Technical:  Can include psychoeducation about the role circadian rhythms can play in bipolar relapse.  Include consideration of relationship between routine and mood for the individual.  Where appropriate, support changes to routine. | Conceptual: in STABILISE work on routine and interpersonal interactions is approached within a broad behavioural framework that also includes consideration of behaviour changes beyond those connected to routine (e.g. rumination whilst engaging in tasks). Also draws upon concepts from DBT such as dialecticism.  Technical:  STABILISE includes concepts and techniques from emotion regulation approaches. Includes concepts and techniques from behaviour therapies more widely. |
| CBT | Conceptual: Draw on behavioural theory of depression; envisage a role for thoughts / mental processes in the onset of maintenance of mood states.  Technical: Include activity monitoring and scheduling, as well as other behavioural techniques including problem-solving, graded task assignment and skills coaching. | Conceptual: STABILISE does not assign a primary role to meaning-making in the onset and maintenance of emotional states; generally views thoughts as mental behaviours; draws upon concepts from emotion regulation therapies, such as dialecticism.  Technical: STABILISE includes wider range of behavioural ideas and techniques than typically feature in CBT (functional analysis, functional equivalence). When working with mental behaviour, predominance of techniques that focus on the utility of the cognitive process rather than its veracity. Includes concepts and techniques from emotion regulation approaches. |
| DBT | Conceptual: Informed by biosocial model, behavioural principles and dialecticism.  Technical: Include skills for understanding and managing emotion states including distress tolerance and emotion regulation skills; include skills for negotiating challenging interpersonal situations. | Conceptual: STABILISE does not necessarily make full use of the range of concepts available in DBT; draws more heavily on behavioural theory of depression and theory underpinning contemporary understandings of bipolar episode vulnerability and maintenance (circadian dysregulation theory, reward system dysregulation theory).  Technical: Greater use of techniques from behavioural activation and IPSRT in STABILISE; does not use full repertoire of DBT techniques; does not use full set of CBT modalities; uses some emotion regulation tools that do not feature in DBT. |
| MBCT | Conceptual: seek to change relationship to internal experience where this is problematic.  Technical: make use of practices in which patient learns to pay attention to present experience non-judgementally and with compassion. | Conceptual: STABILISE does not draw up full theoretical underpinnings of MBCT; also draws upon more “change” oriented approaches.  Technical: STABILISE includes multiple techniques not included in MBCT, particularly “change” techniques; mindfulness-like practices are optional, shorter, and often with multiple intended functions (e.g. relaxation, grounding, practicing attending to one’s needs) in addition to an attentional training function. |
| Behavioural Activation | Conceptual: draw upon behavioural theory of depression.  Technical: STABILISE makes use of all techniques available in behavioural activation for depression. | Conceptual: STABILISE draws upon theory underpinning emotion regulation approaches and in particular DBT.  Technical: STABILISE includes techniques from emotion regulation and in particular DBT. Focus on establishing a sustainable activity pattern rather than increasing activation to reduce depression. Application of functional analysis to “positive” as well as negative mood states. |
