# Supplementary material for: A study protocol for a randomized controlled feasibility trial of behavioural therapy for interepisode bipolar symptoms (STABILISE)
Source: Pilot Feasibility Stud. 2025 Jul 10;11:97. doi: 10.1186/s40814-025-01678-6 (PMC12243248; doi:10.1186/s40814-025-01678-6)
Supplement: Supplementary file 3 — Additional File 3. Means of measuring progress against study objectives. Study objectives with details of how progress or performance against each objective will be measured. [file 40814_2025_1678_MOESM3_ESM.docx]

Additional File 3: Means of measuring progress against study objectives

| Objective | Means of Measurement |
| --- | --- |
| 1. To inform the recruitment and timeline of a future fully-powered trial. | Rates of recruitment from both sites (number of participants randomised per month), presented for each site and for both sites combined. |
| 2. To refine future trial procedures by establishing the acceptability and experience of the trial process to participants, including randomisation and completion of outcome measures. | Outcome measure completion rates including health economics measures, attrition across the two arms, and quantitative and qualitative acceptability data from participants. Descriptive statistics will be presented including within a CONSORT diagram showing participant flow through the study, including number expressing an interest, completing a screening call, attending intake assessment, giving informed consent, being randomised, and completion of each follow-up point. More detailed descriptive statistics showing completion of each measure at each follow-up point will be presented in tabular form. |
| 3. To determine the optimal primary outcome measure in a future trial. | Per-measure completion rates, participant feedback on measures, participant rankings of importance, likely sensitivity to change, and presence of an established minimally important clinical difference value. At this stage, candidate future primary outcome measures include the PHQ9, ALS-SF, brief Quality of Life in Bipolar Disorder Scale (QoL.BD) and Bipolar Recovery Questionnaire (BRQ). Completion rates for each measure will be presented at each time point; participant feedback on measures will be analysed qualitatively. Participant rankings of these four clinical outcome domains plus two additional potential secondary outcomes domains (anxiety and mania symptoms, measured by the Generalised Anxiety Disorder Questionnaire (GAD-7) and Bech-Rafealsen Mania Scale (BRMS) respectively), will be used to provide information on their value to participants: for each domain mean (SD) rank will be presented, where lower score equals higher rank, as well as number (%) of participants selecting each domain as first or second highest-ranked. Rankings will be gathered as part of an embedded study piloting three methods for establishing patient prioritisation of outcomes. In each method participants are asked to indicate the relative importance of change on each of the six outcome areas. For further details of the methods used please contact the corresponding author. To examine sensitivity of the measures to the impact of the intervention, between group mean differences will be calculated (with 95% confidence intervals), as will rates of reliable change on the measures (reliable improvement, reliable deterioration) [1]. The information above will be considered within a stakeholder consensus meeting to agree the optimal primary (or co-primary) outcome measure(s) for a future definitive trial, with the ultimate decision made by the CI. |
| 4. To inform estimation of sample size for a future trial. | Data completeness at follow up (participant attrition) as described for objectives 2 and 3, and standard deviation of candidate primary outcome measures (to compare with reports in published literature). |
| 5. To characterise treatment as usual across individuals and sites. | Data on treatment as usual will be presented descriptively based on health economic data collected at intake and at each follow up point. |
| 6. To further assess the safety and acceptability of the treatment and, based on input from trial participants and clinicians, to further refine and develop the treatment manual and the procedures for training, supervising and assessing the competence of trial therapists | The number of adverse events (AEs) and serious adverse events (SAEs) will be reported descriptively for each treatment group using an As Treated approach, whereby participants will be reported according to treatment actually received, irrespective of group allocation. We will also report the number and percentage of participants experiencing at least one SAE, as well as between group comparisons of number of individuals experiencing an SAE using risk difference and odds ratio with 95% confidence intervals. Acknowledging that AEs and SAEs are more likely to be reported by those in the STABILISE + TAU arm because of regular contacts with their therapist, we will particularly attend to the outcome of between group comparisons of study-related versus unrelated events. Safety will also be assessed by examining rates of reliable deterioration at 30 weeks (number of participants showing reliable deterioration in both arms).  With respect to acceptability this will be evaluated by examining therapy uptake and completion rates: number of participants entering and completing therapy, mean (SD) number of sessions attended and minimum and maximum. It will also be assessed by evaluating quantitative and qualitative feedback from participants and therapists: mean (SD) and minimum and maximum of quantitative ratings on acceptability items; and thematic analysis of participant feedback. The inter-rater reliability of the therapy competence measure will be assessed by two raters independently assessing the same 51 recordings of therapy sessions and their scores being used to calculate the intraclass correlation coefficient using a two-way random effects model, for both consistency and absolute agreement (n=51 allows estimation of ICC of 0.8 with 95% CI of 0.7-0.9). |
| 7. To demonstrate feasibility outside of the lead site by including a second site. | Rates of recruitment and retention across the two sites. |
| 8. To identify, measure and value the cost components for delivering the intervention. | These costs will include any necessary training and materials, as well as staff costs and supervision if necessary. It will take into consideration whether the intervention was delivered face-to-face, online or by phone. |
| 9. To assess the feasibility of collecting health care resource utilisation data and health-related quality of life data using the EQ-5D-5L for obtaining utilities and estimating Quality-Adjusted Life Years (QALYs) at all timepoints. | We will investigate the pattern of missingness as well as identifying which are the key cost drivers on resource utilisation. |
| 10. To examine the feasibility of collecting momentary assessment data at intake, 14 and 30 weeks. | Rates and pattern of missingness as well as through participant report. |
