# Supplementary material for: A study protocol for a randomized controlled feasibility trial of behavioural therapy for interepisode bipolar symptoms (STABILISE)
Source: Pilot Feasibility Stud. 2025 Jul 10;11:97. doi: 10.1186/s40814-025-01678-6 (PMC12243248; doi:10.1186/s40814-025-01678-6)
Supplement: Supplementary file 4 — Additional File 4. Data storage details. Means of storing study data according to data type. [file 40814_2025_1678_MOESM4_ESM.docx]

Additional File 4: Data storage details

| Data format / type | Storage Plan |
| --- | --- |
| Electronic Data | Held by secure data capture platform; stored in secure data storage area (Secure Data Research Hub: SDRH) hosted by University of Exeter. |
| Hard (physical) copies of data | Anonymised and stored in a locked filing cabinet in a locked office in the Department of Psychology, University of Exeter or a locked cabinet in a locked office on local participating NHS Trust premises.  At the end of the study any anonymised paper data will be scanned into electronic form or entered into electronic databases (if not already done). Databases containing data returned by paper and through electronic data capture systems will be merged with assistance from Exeter Clinical Trials Unit prior to data analysis. Range checks will be used to help identify erroneous datapoints prior to analysis. Resulting files will be stored in the secure data storage area of the University of Exeter and the original paper copies and audio-recordings destroyed confidentially. |
| Hard copies of consent forms | Locked cabinet separately to data. |
| Audio recordings for research purposes | Secure data research hub, University of Exeter. |
| Data collected as part of delivery of therapy, forming part of the NHS patient record. | In accordance with the Information Governance requirements of their service. Within NHS services providing the therapy, participant information will not be stored anonymously as it will form part of the patient record. |

**Additional References**

1. Jacobson NS, Truax P. Clinical significance: a statistical approach to defining meaningful change in psychotherapy research. Journal of Consulting and Clinical Psychology. 1991; 59:12-19.
